# The role of legal medicine professionals in preventing pregnancy and sexually transmitted infections among female victims of sexual assault

**DOI:** 10.1007/s12024-023-00578-6

**Published:** 2023-03-07

**Authors:** Renjulal Yesodharan, Pratibha Kamath, Vishnu Renjith, Nirmal M. Krishnan, Udara Dilrukshi Senarathne, Suja Kumari Sasidharan, Tessy Treesa Jose, Vinod C. Nayak

**Affiliations:** 1 Department of Psychiatric Nursing, Manipal College of Nursing, Manipal Academy of Higher Education, Manipal, Udupi, Karnataka India; 2 Department of OBG Nursing, Manipal College of Nursing, Manipal Academy of Higher Education, Manipal, Udupi, Karnataka India; 3 School of Nursing & Midwifery, Royal College of Surgeons in Ireland, 123 St Stephen’s Green, Dublin 2, Ireland; 4 Department of Forensic Medicine and Toxicology, Kasturba Medical College, Manipal Academy of Higher Education, Manipal, Udupi, Karnataka India; 5 Faculty of Medical Sciences, Department of Biochemistry, University of Sri Jayewardenepura, Nugegoda, Sri Lanka; 6 Department of Obstetrics and Gynaecological Nursing, Amrita College of Nursing, Amrita Vishwa Vidyapeetham, Ernakulum, Kerala India

**Keywords:** Contraception, Crime victims, Forensic nursing, Forensic sciences, Post-exposure prophylaxis, Pregnancy, Preventive health service, Sex offences, Sexually transmitted diseases

## Abstract

Sexual violence can have an overwhelming impact on the victim’s physical and mental health; the consequences include unintended pregnancy and sexually transmitted infections (STIs). Therefore, the examiners must assess victims for possible pregnancy and sexually transmitted infections as a part of the sexual assault examination. This article aims to orient the medico-legal examiners towards their role in preventing unintended pregnancy and sexually transmitted infections among victims of sexual assault. Prompt detection of pregnancy or STIs is critical, as any delay would adversely affect the successful administration of emergency contraception and post-exposure prophylaxis (PEP) against human immunodeficiency virus (HIV) and other sexually transmitted infections.

## Background

The overwhelming effect of sexual assaults (SA) is short, but the long-term physical and psychological consequences can be more deleterious. These consequences include pregnancy, sexually transmitted infections (STIs), and reproductive tract infections [1, 2]. Therefore, the medico-legal professionals attending to female sexual assault victims (SAVs) must assess for the possibility of pregnancy and STIs as a part of the sexual assault examination [3]. The examination and subsequent medical support should include assessing the victim for pregnancy and STIs, care for victims, informed consent for treatment, emergency contraception, follow-up care, etc. [4–6]. It is also mandatory that an experienced clinician conducts the examinations of survivors of sexual assault in a way that minimises further trauma.

Unintended pregnancy as a consequence of sexual assault has dire impacts on the health and socioeconomic well-being of the victims and their loved ones. Although the risk of conception after an unprotected sexual encounter (UPSE) may be low and is based on several factors [7], the socioeconomic impact of rape-related pregnancy (RRP) could be devastating. Therefore, RRP is considered a public health concern that adds to the adverse outcomes of sexual assaults [8] and, therefore, needs to be assessed at the earliest with appropriate measures taken for prevention. Furthermore, unintended pregnancy is the main reason for abortions [9], which may again add to the adverse outcomes of RRP. STIs are another significant consequence of sexual assault [10, 11]. Therefore, SAVs need multidisciplinary care, which include assessing and managing STIs [12, 13]. Even the presence of STIs and the isolation of the causative organisms are strong indicators of child sexual abuse [14]. STIs are generally caused by bacteria, viruses, protozoa, and fungi and can be transmitted through body fluids or by direct contact with the skin.

Assessing for pregnancy and STIs is very critical as any delay in the diagnosis would affect the successful administration of emergency contraceptives (EC) and post-exposure prophylaxis (PEP) against HIV and other STIs [15]. Therefore, this paper aims to orient medico-legal professionals about their role in preventing and managing pregnancy and STIs among the SAVs.

## Main text

### I. Prophylaxis against pregnancy

#### Probability of pregnancy: considerations

The probability of getting pregnant after sexual assault is dependent upon various factors, some of which are discussed below.

#### The type of sexual assault

Contact of seminal fluid with the vagina of the victim poses a high risk of pregnancy. The unprotected vaginal penetration by the penis is always on top of the list. Sodomy will also become a risk if the seminal fluid flows from the anus to the vagina. An additional case that needs to be considered is the perpetrator ejaculating near the vagina.

#### The victim’s menstrual cycle and the life span of the ovum and sperm

The exact duration of the menstrual cycle varies for each woman. The average menstrual cycle is considered to be 28 days. In some cases, menstrual cycles can be shorter than 21 days or longer than 41 days, during which the period can be regular and normal. Ovulation occurs approximately on the 14th day of the menstrual cycle. The life span of an ovum is about 24–48 h, whereas the life span of sperm is about 4–5 days. These gamete characteristics mean that the highest risk of conception following a single UPSE is from days 9–16 of the menstrual cycle (4.9 to 9.7%) [16]. Li, Wilcox, and Dunson (2015) also estimate that the risk of pregnancy after a single UPSE, irrespective of the day of the menstrual cycle, is about 9.7%. They further observed that the highest probability of pregnancy was on the 13th day of the menstrual cycle. The probability of becoming pregnant is nearly zero (0.001–0.002) in the initial 3 days of the menstrual cycle. Women with irregular menstruation have a higher probability of getting pregnant in the latter part of the cycle.

#### Use of condoms by the perpetrator

A study conducted by O'Neal et al. found that only 11.7 to 15.6% of perpetrators use condoms [17]. Not using a condom, any damage to the condom, or the condom slipping off during the assault increases the risk of pregnancy.

#### Use of contraceptive methods by the victim/perpetrator

- **Sterilisation and long-acting reversible contraception (LARC)**
  LARC methods provide birth control measures for both the perpetrator and the victim. This includes vasectomy for the perpetrator and a variety of methods such as tubectomy, hysterectomy, Nexplanon, progesterone-based intrauterine devices (IUDs), copper IUDs, and non-hormonal IUDs for the victim. The rate of pregnancies after using LARC is lower when compared with oral contraceptives. However, tubal ligation had demonstrated a failure rate of 2.6 per 100 women [18].
- **Combined oral contraceptive pills, progesterone pills, and depot preparations (shots)**
  Oral contraceptive pills contain either progestin or a combination of oestrogen and progestin. The combination of oestrogen and progestin inhibits the release of luteinising hormones and follicle-stimulating hormones from the pituitary gland. Progestin inhibits ovulation and changes the mucus surrounding the ovum, making it difficult for sperm to penetrate. The circulating serum levels of exogenous oestrogen or progesterone that interfere with their action at a cellular level determine the efficacy of oral contraceptives. Inadvertent pregnancies (6–9%) are not uncommon in combined pill users and are usually due to poor compliance [19, 20].
- **Condom for men and women**
  The pregnancy rate among men or women who used condoms is 13–21% [21].

#### Perimenopausal women

Perimenopause is the time around menopause, starting a few years before menopause and ending 12 months after the last menses. As a result, the pregnancy rate is considerably low, although perimenopausal women can also become pregnant [22, 23].

#### Special population (transgender men)

Transgender men (female to male) also risk pregnancy with unprotected sexual intercourse if they do have not had a sterilisation procedure or achieved menopause [24].

#### The use of emergency contraception

Pregnancy associated with sexual assault can be prevented using emergency contraception (EC). It can prevent over 95% when taken within 5 days after the unprotected sexual encounter. However, emergency contraceptives should be taken only in cases of emergency and not as an alternative to regular contraception. The most effective method is a copper intrauterine device (Cu-IUD), which can be left in place as ongoing contraception afterwards. Selective progesterone receptor modulators such as ulipristal acetate are among the most effective emergency contraceptive pills (ECPs) available [25]. ECPs containing levonorgestrel are another method of EC. The details of the ECs are mentioned in Table 1.

**Table 1:** Emergency contraceptive methods and their action, failure rate, the window of efficacy, and advantages

**Emergency contraceptive method and dose**	**Mechanism of action**	**Failure rate (occurrence of pregnancy after the use of EC)**	**The window of efficacy after an unprotected sexual encounter (UPSE)**	**Advantages over other ECs**
Copper IUD	• The toxic effect on sperm and ova • Harmfully affect motility and viability of sperm • Adversely affect viability and transportation of ova • It prevents implementation by a local endometrial inflammatory reaction	< 1%	It can be inserted within 5 days after the UPSE in a cycle or within 5 days of the earliest estimated date of ovulation, whichever is later	• Copper IUD is the only method that is effective after the starting of ovulation • Copper IUD is not affected by BMI of the individual or action of any drugs
ECPs containing ulipristal acetate (30 mg single oral dose)	• Delays ovulation for at least 5 days until sperm from the UPSE is no longer viable (It delays ovulation even after the start of the luteinising hormone (LH) surge)	1.3–2.1% Fine et al*.* [52]	Within 3–5 days after UPSE Glasier et al*.* [53]	• UPA–ECP is the only oral EC that is expected to be effective if UPSE took place 96–120 h ago (LNG-ECP is ineffective if taken more than 96 h after UPSE)
ECPs containing Levonorgestrel (LNG) (1.5 mg single oral dose or 3 mg when the woman is taking the enzyme-inducing drug or has a BMI of more than 26 kg/m^2^)	• If taken before the start of the LH surge, it delays or prevents follicular rupture and causes luteal dysfunction • Delays ovulation for at least 5 days until sperm from the UPSE is no longer viable	0.6–2.6%	Within 72 h after UPSE	• LNG-ECP is effective even if a woman has recently taken progestogen (The effectiveness of UPA-EC could theoretically be reduced in this case)

#### Follow-up care and diagnosis of pregnancy

In cases of sexual assault, a follow-up visit to a healthcare facility is essential. A visit within 2 to 4 weeks after the initial examination can help the victim be tested for possible pregnancy and get early termination of the pregnancy if needed. A blood and/or urine sample analysis is required for pregnancy testing. A pregnancy test within 5 days after sexual assault may show positive but does not necessarily indicate that the victim became pregnant because of assault.

The accuracy of different pregnancy tests varies depending on the assay method, the specimen, and the date of specimen collection. The freely available urine beta-hCG strip method is a point of care testing (POCT) device based on lateral-flow immunochromatography. It qualitatively detects the presence of beta-hCG in urine specimens at 20 mIU/mL sensitivity in most cases [26]. It should be noted that false-negative pregnancy results may occur in very high urine beta-hCG concentration due to the ‘high-dose hook effect’ with the assay method, which can be overcome by testing diluted urine [27]. The serum beta-hCG analysis is a quantitative immunoassay method with 97% and 100% specificity [28].

#### Medical termination of pregnancy (MTP)

SAVs with unwanted pregnancies may choose to have induced abortions. The principal determinants of the safety of induced abortions are the gestational age at abortion and the method used. The earlier the MTP is performed, the safer the procedure is. The lowest risk is when the procedure is performed 7–10 weeks after the last menstrual period. Induced abortions are restricted by law in a few regions of the world, while safe abortion techniques are legalised and practised in most countries [29]. The legalisation of abortions reduces the chances of illegal and unsafe practices. For example, the Government of India amended the Medical Termination of Pregnancy Act, 1971 [30] and established rules giving power to the medical board to allow or deny termination of pregnancy beyond 24 weeks of the gestation period. Medical termination of pregnancy can be attained by administering mifepristone combined with a suitable prostaglandin, such as misoprostol or a prostaglandin analogue [31]. If the products of conception remain within the uterus, a manual vacuum aspiration can be conducted under local anaesthesia on the cervix head. Intrauterine injection of hypertonic solutions, such as saline or urea, augmented by oxytocin, prostaglandin, laminaria, or some combination of these can also be used for MTP. The other methods of MTPs are extra-amniotic injections of ethacridine lactate and surgical procedures such as dilatation and evacuation (D&E).

#### Pre- and post-procedure counselling in cases of MTPs

Counselling is a continuous, structured, and systematic interaction in which an individual voluntarily receives emotional support and guidance from a competent person in an environment that is helpful for to open sharing of thoughts that influences health and well-being [32]. The main focus of counselling before and after a medical termination of pregnancy is to reduce the stigma around the issue by normalising it. An adolescent who has an unwanted pregnancy or an STI may feel embarrassed or ashamed. Therefore, it is essential that counsellors actively minimise the stigma associated with the issue by stating to the victim, ‘I have treated several people with the same problem you have’ [31, 33].

#### Medico-legal aspects of MTPs

Globally, the legality of abortion can be grouped into five categories.

- All types of abortions are prohibited or illegal.
- Unlawful abortions are prohibited and penalised, but no additional information on lawful abortions.
- Allows or permits abortion only on one or more legal grounds.
- Entitles a woman for abortion on request with no requirement for justification.
- Abortion is regulated only as a health intervention in the health system [29].

Abortion laws and policies vary across countries and regional jurisdictions. On a global scale, the World Health Organization has laid out specific standard for abortions [29, 32, 33]. These guidelines recommend that MTP be performed by a specialised doctor with postgraduate clinical training and a specialisation in obstetrics and gynaecology and reiterate the need to obtain consent from the individual undergoing MTP. Additionally, it is a best practice to ensure the presence of a female health care professional during the examination and the MTP procedure.

#### Aborted foetal material (abortus evidence)

Abortus will be assessed to identify the biological parents using deoxyribonucleic acid (DNA) typing after the voluntary interruption of pregnancy [34–36]. The aborted material consists of the placenta, which contains maternal and foetal sections. The foetal DNA will be isolated from the aborted material and a foetal profile will be obtained through repeated sampling. Chorionic villi samples usually give a ‘clean’ or ‘nearly clean’ foetal DNA profile without maternal DNA contamination. However, excess maternal DNA can hinder the detection of foetal genotypes [36

### II. Prophylaxis against sexually transmitted infections

#### Factors affecting STI risk

The actual risk of contracting STIs depend on many factors like the prevalence of STIs in the population, the type of sexual activity performed, the use of condoms, the level of immunity, and the rate of serial monogamy in society. STIs are common in young individuals due to the presence of columnar epithelium on the ectocervix (cervical ectropion) [37]. Condoms may help in preventing STIs which are transmitted through seminal or vaginal fluids (chlamydia and gonorrhoea), but are less effective in preventing STIs that are transmitted through sores or cuts on the skin (genital herpes, syphilis, and human papillomavirus).

#### Barriers to assessing STI risk

Individuals may be afraid to be tested for STIs to avoid revealing pre-existing infection and previous sexual activities. The prior use of antibiotics (prescribed either for any other condition or over-the-counter medication) can also mask any STIs that may be present otherwise. Underestimating the risk of STIs is also another barrier effectively assessing STI risk [38].

#### Types of STIs

More than 30 different infectious agents are known to cause STIs. Out of these, eight pathogens cause major STIs. These include curable infections such as syphilis, gonorrhoea, chlamydia, and trichomoniasis, as well as incurable infections such as hepatitis B, herpes simplex virus (HSV), human immunodeficiency virus (HIV), and human papilloma virus (HPV). Globally, more than a million cases of STIs are reported daily. In 2020 alone, the WHO estimated 374 million new cases of curable STI around the globe, with chlamydia being the highest, amounting to 129 million cases, followed by gonorrhoea (82 million), syphilis (7.1 million), and trichomoniasis (156 million) [39–41]. The details of curable STIs are mentioned in Table 2.

- **Chlamydia**

**Table 2:**
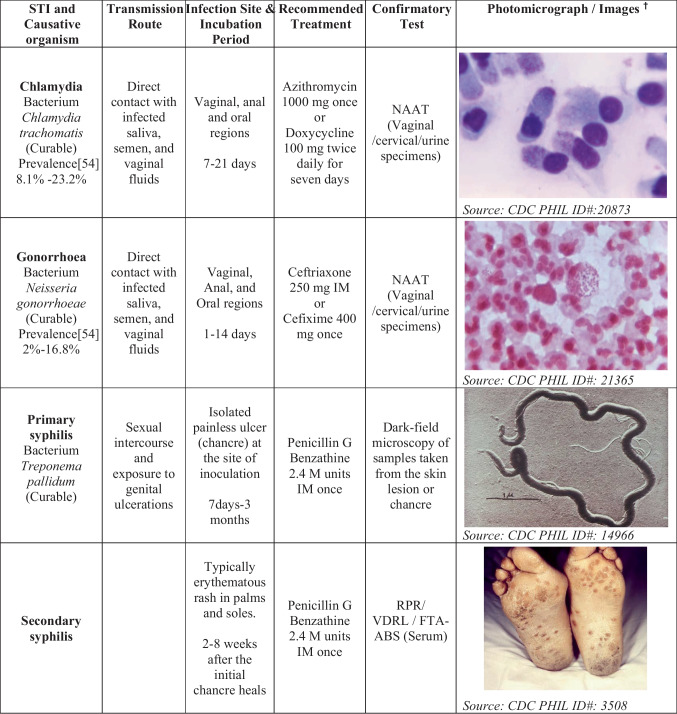

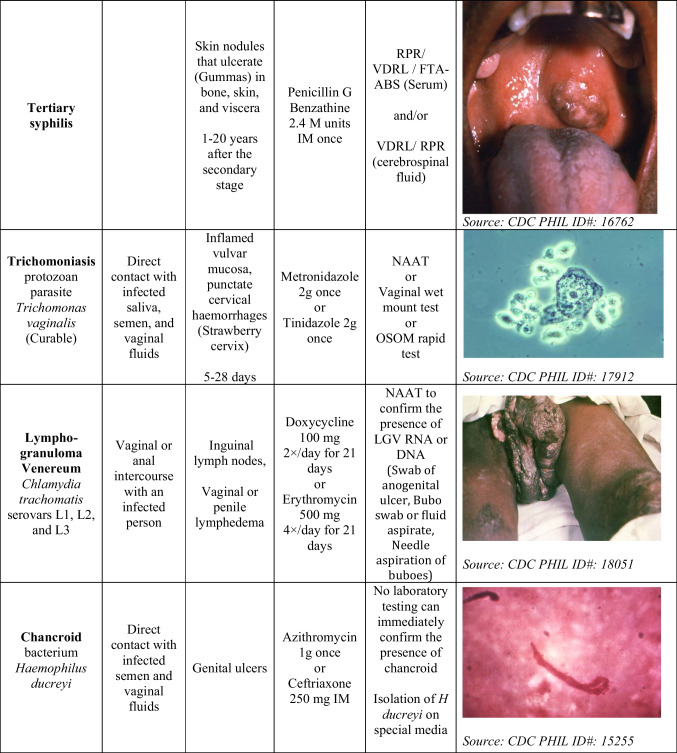
STIs and their transmission route, infections site, incubation period, recommended treatment, confirmatory test, and images

M units—million units**,** IM**-** intramuscularly

^**†**^ *Photos adopted from Public Health Image Library (PHIL) maintained by CDC*

Globally, chlamydia is the most prevalent bacterial STI [41]. The infection can be asymptomatic or show various symptoms, such as pelvic pain, rectal pain, dysuria, vaginal discharge, and a sore throat [39]. The severity of the symptoms is usually subjective to the level of infection and the route of exposure. These asymptomatic infections can be spread inadvertently by the infected person to others [13].

(b) **Gonorrhoea**

Gonorrhoea also remains a major global public health concern. Patients with gonorrhoea can be asymptomatic or present with localised infection, showing symptoms such as pelvic pain or rectal pain, joint pain, penile or vaginal discharge, or sore throat [39].

(iii) **Syphilis**

*Treponema pallidum* is a spirochete responsible for syphilis, which spreads from individual to individual by direct contact with genital ulcerations or through sore (chancre). These sores can occur in, on, or around the vagina, penis, rectum, anus, and mouth or lips. Syphilis can spread during oral, anal, or vaginal intercourse with an infected person.

(iv) **Trichomonas**

Trichomonas is the most widespread protozoan STI caused by *trichomonas vaginalis*. The initial symptoms start within 3–28 days. For a few women, the infection may be asymptomatic. Epididymitis urethritis and prostatitis are the common symptoms present in men, whereas women may show vaginal discharge, dysuria, or vulval irritation. Women may also have vulvar erythema and punctate cervical haemorrhages, described as a ‘strawberry cervix’.

(e) **Human immunodeficiency virus (HIV)**

HIV is a viral infection caused by an enveloped ribonucleic acid (RNA) virus that may be passed through sexual contact and sharing syringes and/or sharing needles with an infected person. Various factors increase the risk of transmitting HIV including bite injuries caused by the offenders, multiple offenders, anal and vaginal penetration, occurrence of genital trauma, anal or vaginal tear, the presence of sperm or semen in/around the genital area, and the offender(s) being injection drug user(s).

(f) **Hepatitis B virus (HBV)**

HBV is a hepatotropic virus transmitted through the serum, semen, and vaginal fluids [42]. HBV is 50 to 100 times more virulent than HIV and can cause severe liver diseases, including acute and chronic hepatitis, cirrhosis, and hepatocellular carcinoma [43].

#### Prevention counselling

Counselling related to STIs, especially symptoms, treatment options, and care after discharge from the hospital, is essential. The risk associated with STI exposure and the potential risk of transferring to future or current consensual partners also needs to be discussed with the SAVs. High psychological distress can be alleviated by giving proper information about the follow-up assessment and care [44].

#### Screening for STI

STI screening is advised between 3 and 7 days and 2 weeks after the assault (as dictated by incubation periods of *Neisseria gonorrhoeae* and *Chlamydia trachomatis*) or later if prophylactic antibiotics have been taken.

#### Protocol for testing

- **Nucleic acid amplification test (NAAT)**

*Neisseria gonorrhoeae* and *Chlamydia trachomatis* can cause asymptomatic infections and the diagnosis based on the symptoms alone is not an effective approach to treatment. In contrast, NAAT can be adopted for a more accurate diagnosis. The sample can be collected from urine in both sexes or by swabbing the vagina or cervix in women. Pharyngeal or rectal swabs can also be considered depending on the case [45]. NAAT is extremely sensitive and specific for diagnosing the existence of *Chlamydia trachomatis* (sensitivity: 97.5–98.5 and specificity: 98.4–99.4) and *Neisseria gonorrhoeae* (sensitivity: 98.0–98.5 and specificity: 99.6–99.8) [46].

(b) **Cervical cytology**

In a conventional cervical cytological test, the sample is spread on a thin film over a small area onto a dry slide and examined under the microscope. This test is typically conducted 10–20 days after the last menstrual periods, whereas a liquid-based cytology can be executed at any time during the menstrual cycle. Some women may present a mucopurulent discharge and the test can be done after clearing the discharge with a cotton swab soaked with saline. Using a cytobrush to sample the cervical transformation zone improves the accuracy of the cytological test [40].

(iii) **Dark-field microscopy**

Dark-field microscopy is a sensitive and specific imaging technique for demonstrating *T. pallidum* by creating a contrast between the object and the adjacent field so that the background is dark and the object is bright. It is the most specific and sensitive technique to diagnose syphilis when an active chancre or condyloma lata is present, as it allows for a presumptive diagnosis of syphilis even before the development of antibodies. Thus, a dark-field microscopy has the advantage of detecting infection early and on-site, which helps in early identification and treatment, which indirectly helps in containing the spread of infection [47, 48].

(iv) **Serological testing**

HIV, HBV, and syphilis infections can be detected using serological testing by analysing the presence of antibodies (Ab) or antigens (Ag). An antigen or antibody test can usually detect HIV infection between 18 and 45 days after exposure, whereas a NAAT can usually detect an HIV infection between 10 and 33 days after exposure. The hepatitis B surface antigen (HBsAg) is the first serologic marker appearing in the serum between 6 and 16 weeks following exposure to the HBV, which can be used for the diagnosis of HBV infection. If HBsAg and anti-HBc (hepatitis B core Ab) are present in the absence of IgM anti-HBc, this can be used to diagnose a chronic HBV infection. Screening for syphilis can be done by detecting Abs against *T. pallidum* using the venereal disease research laboratory (VDRL) test and can be confirmed by *Treponema pallidum* particle agglutination assay (TP-PA).

#### Empiric antimicrobial regimen for bacterial STIs

The Center for Disease Control and Prevention (CDC) recommends a single dose of Ceftriaxone 500 mg/IM (if weight weighing ≥ 150 kg, 1 g of Ceftriaxone) with doxycycline 100 mg two times a day orally and metronidazole 500 mg two times a day orally for 7 days as empiric antibiotics for sexually transmitted bacterial infections [40]. However, the efficacy of these regimens in preventing infections after a sexual assault has not been evaluated. Therefore, if the victim requires specific treatment, the organism needs to be identified.

#### Non-occupational post-exposure prophylaxis (PEP) against HIV

Prevention of HIV infection after a possible exposure can be achieved by administering antiretroviral therapy (ART) within 72 h (3 days). PEP should be used only in emergencies and not meant for regular use [49]. The PEP should be given to SAVs who are HIV negative or do not know their status. The effectiveness of the regimen is higher when administered early after the potential exposure. A rapid fourth-generation HIV testing preferably needs to be done before the initiation of PEP and a negative result requires immediate starting of PEP. If such fourth-generation tests are not available, then PEP can be started and the samples can be sent to labs for confirmation. Upon a positive result, the PEP regimen is stopped, starting of therapeutic regimen [49–51], The PEP regimen and alternative treatment regimen recommended by CDC are given in Table 3.

**Table 3:** PEP regimen and alternative treatment regimen recommended by CDC

**Population**	**PEP regimen(preferred)**	**Alternative regimen**
Adults and adolescents (≥ 13 years) with normal renal function (Cr _CL_ ≥ 60 mL/min)	Tenofovir disoproxil fumarate 300 mg once daily or emtricitabine 200 mg once daily + Dolutegravir 50 mg once daily or Raltegravir 400 mg twice daily	Tenofovir disoproxil fumarate 300 mg once daily or emtricitabine 200 mg once daily + Darunavir 800 mg (as two, 400-mg tablets) once daily and ritonavirb 100 mg once daily
Adults and adolescents (≥ 13 years) with renal dysfunction (Cr _CL_ ≤ 59 mL/min)	Zidovudine and Lamivudine, with both doses adjusted to degree of renal function + Raltegravir 400 mg twice daily + Dolutegravir 50 mg once daily or Raltegravir 400 mg twice daily	Zidovudine and Lamivudine, with both doses adjusted to degree of renal function + Darunavir 800 mg (as two 400-mg tablets) once daily + Ritonavirb 100 mg once daily
Children aged 2–12 years	Tenofovir DF, emtricitabine, and Raltegravir, with each drug dosed to age and weight	Zidovudine and Lamivudine + Raltegravir or lopinavir/ritonavir + Raltegravir (lopinavir/ritonavir dosed to age and weight)

*Cr *_*CL*_, *creatinine clearance*

#### Risk assessment for PEP

- There is substantial risk for HIV acquisition when SAVs had exposure to semen, rectal secretions, vaginal secretions, breast milk, blood, or any body fluids that are visibly contaminated with blood on the non-intact skin, mouth, eye, vagina, penis, rectum, or another mucous membrane, or percutaneous contact from a known source of HIV.
- There is negligible risk for HIV acquisition when SAVs had exposure to urine, nasal secretions, saliva, sweat, tears without any visible blood on the non-intact skin, mouth, eye, vagina, penis, rectum, or another mucous membrane, or percutaneous contact from a from a known or suspected source of HIV.

A PEP regimen needs to be started if there are signs of substantial risk, provided such exposures occur within 72 h [49, 50].

#### PEP regimen (CDC)

A 28-day course of PEP is recommended for HIV-uninfected persons who have a substantial risk for HIV acquisition within 72 h [50].

#### Prophylaxis against hepatitis B

SAVs who are not previously vaccinated or partially vaccinated need to administer a single dose of the hepatitis B vaccine. If the perpetrator is available for examination and found to be HBsAg positive, then the SAV who was not vaccinated prior should receive the hepatitis B vaccine and hepatitis B immunoglobulin during the initial medico-legal assessment. Follow-up vaccines should be administered during 1–2 months and 4–6 months after the initial dose. If the SAV has been vaccinated before, then a single booster dose of the hepatitis B vaccine needs to be administered [40, 50].

#### Prophylaxis against human papilloma virus

The HPV vaccine can be administered to SAVs (9–21 years for males and 9–26 years for females) at the time of the initial medico-legal examination and two follow-up doses need to be administered 1–2 months and 6 months after the first dose [40].

#### Counselling for individuals taking PEP

The counselling sessions should primarily focus on coping strategies for the victimised individual. Motivational interviewing and training of adaptive coping skills can be considered part of the counselling sessions. PEP can reduce the risk of infection, but it is not always effective. Counselling also needs to focus on the protective behaviour of the individuals with their sex partners. Adherence counselling can also separately be provided to the individuals [49, 50].

## Conclusion

SAVs have a higher risk of becoming pregnant and acquiring STIs, which can cause a severe impact on their physiological, psychological, and social functioning. Consequently, it also affects their sense of self, health, and well-being. Thus, the early detection of STIs and pregnancy is very critical, and any delay would adversely affect the successful administration of EC and PEP against HIV and other STIs.

## Key points

1. The overwhelming effect of sexual assault is short, but the long-term physical and psychological consequences can be more harmful.
2. Unintended pregnancy as a consequence of sexual assault has dire impacts on the health and socio-economic well-being of the victims and their loved ones.
3. Promptness in the diagnosis of pregnancy and sexually transmitted infections is very critical as any delay would affect the successful administration of emergency contraceptives and post-exposure prophylaxis against HIV and other STIs.
4. Pregnancy-associated with sexual assault can be prevented using emergency contraception (over 95%) when taken within five days after the unprotected sexual encounter.

## Abbreviations

*EC*: Emergency contraception
*ECP*: Emergency contraceptive pill
*HBV*: Hepatitis B virus
*HPV*: Human papilloma virus
*IUD*: Intrauterine devices
*LARC*: Long-acting reversible contraception
MTP: Medical Termination of Pregnancy
*NAAT*: Nucleic acid amplification test
*PEP*: Post exposure prophylaxis
*RRP*: Rape-related pregnancy
*SA*: Sexual assault
*SAV*: Sexual assault victim
*STI*: Sexually transmitted infection
*UPSE*: Unprotected sexual encounter
